# Taking advantage of between- and within-participant variability?

**DOI:** 10.3389/fpsyg.2014.01235

**Published:** 2014-10-29

**Authors:** Cyril Perret, Sonia Kandel

**Affiliations:** ^1^CeRCA, Centre National de la Recherche Scientifique, Unités Mixtes de Recherche 7295, Université de PoitiersPoitiers, France; ^2^Laboratoire de Psychologie et NeuroCognition, Centre National de la Recherche Scientifique, Unités Mixtes de Recherche 5105, Université Grenoble AlpesGrenoble, France

**Keywords:** central tendency effect, statistical distributions, between-participants variability, within-participant variabitity, mixed-effects models

⟪ All other things being equal or held constant ⟫ (i.e., *ceteris paribus*). This experimental principle is probably one of first concepts that teachers present in methodology courses in universities all around the word. Studying the influence of one (or more) experimental factors on a specific dependent variable requires an adequate control of all other sources that might affect it. This epistemological position is directly derived from positivism (e.g., Comte, 1869, 2010). According to this view, the most important scope in science is to develop theories that describe and model the environment and at the same time exclude all micro-variations. In other—philosophical—words, science attempts to understand what remains invariant despite constant transformation of the world. Language sciences follow this principle (psycholinguistics, linguistics, neurobiology of language processing, among others). Most studies try to differentiate the characteristics that human beings share—i.e., universals—from what is individual or specific.

Stochastic between- and within-participant factors are generally considered in experimental setups. However, this control is done more by habit than with a deep meditation on the way they can affect the outcome. Individuals are indeed specific. In a word reading task for instance, the Reaction Time variation across participants follows a Gaussian distribution. Moreover, a participant never performs identically when repeating the task. Individual performance is systematically subject to micro-variations across a set of similar items. In language science experiments, researchers diligently follow methodological recommendations. They generally recruit a group of 20–30 participants and select samples of homogeneous items for each experimental condition. In both, the researchers hope—or at least expect—to have representative samples. This is essential for the elaboration of models with different sources of variability. That is, it is possible to separate random influences from fixed-effects.

Experimental samples are then done to deal with the “fear” of between- and/or within-participant variability. Nevertheless, as in many nightmares, this fear is not totally rational. In their article, Libben et al. (2014) “*Psychocentricity and participant profiles: Implications for lexical processing among multilinguals*” presented a good example. The authors proposed a tool that takes into account the high diversity of multilingual participant profiles. The linguistic experience for each language is specific for every multilingual individual. So the problem when studying multilingualism is the stability of linguistic representations in each participant's mental lexicon. Each individual's language system is specific because he/she has different environmental inputs/outputs for each language. As multilingual person's languages often serve different social and communicative purposes, they form a unique whole that differs qualitatively from a monolingual's system. In other words, a bilingual's language system is not two monolingual systems in one brain (Grosjean, 1989). Another problem is the relation between language production and comprehension. Monolinguals show comparable abilities in the two modalities. This does not always hold for multilingual people. In sum, the difficulties Libben et al. (2014) highlighted focus on the consideration of between-participant variability and its modeling. Most studies rely on a quite monolithic conception of the participants' multilingualism. They do not take into account adequately the variability of the participants' characteristics and skills. Again, the latter do not behave like two or more monolinguals in every language they speak (e.g., Montrul and Sánchez-Walker, 2013). This means that in psycholinguistic studies on multilingualism experimental groups are by definition highly heterogeneous. Heterogeneity within the group is a real problem because it produces random “noise.”

In more general terms, the authors raise the issue of the so-called central tendency. It consists of privileging a position parameter (e.g., mean, median, etc.) instead of its distribution. The idea is that this parameter is the best approximation of its distribution. It allows for an adequate control of the random error influence. The latter can be considered as a stochastic phenomenon. Therefore, a group's specific behavior would yield a sampling of the random error in agreement with its probability distribution. Moreover, the mean of the random error influence tends to zero when the number of observations that form the distribution increases. As a result, the position parameter that is calculated from the data distribution of an adequate sample should not be influenced by the random error. In language science, we use central tendency rather systematically. The more frequent one is the analyses by participants (F_1_) and items (F_2_) (e.g., Forster and Dickinson, 1976; Raaijmakers, 2003) derived from Clark's (1973) proposal. The idea of checking F_2_ is to verify whether a significant fixed-effect exists when the random effects variable of the statistical analysis corresponds to the experiment's items. So for each item we calculate the mean of the participants' responses. This implies that the random error resulting from the between-participant variability is captured perfectly from the participants' responses. In other words, participant samples have to allow for the estimation of between-participant random error.

Although Libben et al. (2014) did not directly discuss this issue, the central point is the ability to estimate correctly the random error from small samples that are typical in language sciences. The major advantage of the profile method called “psychocentricity” is indeed that it models better between-participant variability than the use of central tendency of multilingual characteristics. We can take another example derived from the idea of central tendency, namely lexical frequency. This is one of the most well-known independent variables that affect linguistic behavior. When researchers were looking for evidence in support of the idea that a word is stored in the mental lexicon, they tried to show facilitation in the processing of the items that occurred or were used more frequently. This is widely known as the lexical frequency effect. These experiments employed words for which mean frequency values were available from databases such as Lexique2 for French (New et al., 2004) or CELEX for English, Dutch and German (Baayen et al., 1993). However, it is possible to question the validity of this measure. Is the personal experience of the individuals participating in the study equivalent to what the frequency value of the table denotes? The difference between the theoretical value and the actual experience of the participant with the word can be a source of random noise. We can easily assume that these differences should be extremely variable among participants. A sample must be recruited to estimate correctly this within-participant random error but, as with multilinguals, 20–30 participants seems to be a too small sample for this purpose.

The central tendency principle (e.g., F_1_/F_2_) has been used almost systematically for a long time mainly because of statistical constraints. In language sciences, researchers used statistical models that did not allow to simultaneously take into account the variability across participants and items (e.g., Forster and Dickinson, 1976; Raaijmakers, 2003). The tools to go beyond the analysis of central tendency only appeared in the last 20 years. As Libben et al. (2014) pointed out, the development of non-linear and linear mixed-effects models allowed to examine a larger spectrum of situations (e.g., Pinheiro and Bates, 2000; Baayen et al., 2008; Quené and van der Berg, 2008; Bar et al., 2013)^1^. The basic idea underlying these models is to avoid fixing a priori constraints on the characteristics of the variance/covariance matrix. For instance, why work under a variance homogeneity assumption when heteroskedasticity can be modeled? The analysis is conducted on the whole data set (one value per participant/item pair) and not from a central tendency (e.g., Pinheiro and Bates, 2000; Baayen et al., 2008; Quené and van der Berg, 2008; Bar et al., 2013). The main advantage of the mixed-effects model is the freedom to deal with the sources of random variability (within- and between-participants). Rather than trying to collect (hopefully) adequate samples to estimate random error, the specific characteristics of the variance/covariance are directly modeled. Moreover, the specific profile of a multilingual participant or specific frequency of exposures can be modeled directly in the statistical analysis.

Moreover, mixed-effects models can take into account a final point that is directly related to the source of random variability. These models are called mixed because they are used to model interactions between fixed effect variables (e.g., the independent variable/s of the study) and random effect variables (e.g., participants and items). This is particularly relevant when we want to explore between-participant variation of a fixed effect. For example in picture naming experiments, it is well known that the participants begin to articulate the name of the image much faster (about 300 ms) than to write it (e.g., Bonin et al., 1998). Perret and Laganaro (2013)^2^ provided evidence indicating that this result is in fact due to different initialization criteria between the two tasks. The point we would like to make is that they also observed a mixed-effect between the production mode (oral vs. written) and the participants' random effect variable. This means that the criterion for picture naming responses was modality dependent but also that it is specific to each participant. Without mixed-effect models, we could not have explored this hypothesis.

To conclude, we believe that Libben et al. (2014) provide an original tool that seems very promising. It seems more profitable to include within- and between-participant variability in the statistical model than controlling them with position parameters computed from (larger?) samples. Although the psychocentricity perspective (within- and between-participant) increases the complexity of the psycholinguistic enterprise, it opens the possibility to individualize concepts of language analyses. The examples we presented are far from being exhaustive. However, they support the idea that modeling within- and between-participant variability precisely is one way to explore the invariants of human cognitive functioning.

## Conflict of interest statement

The authors declare that the research was conducted in the absence of any commercial or financial relationships that could be construed as a potential conflict of interest.

## Footnotes

^1^Our goal is not do describe this kind of model. We refer to the special issue on modeling in language science that appeared in the Journal of Memory and Language (2008; number 59).

^2^See Alario and Moscoso del Prado Martin (2010) for an example of mixed-effect by items.
